# A Survey on AI-Driven Digital Twins in Industry 4.0: Smart Manufacturing and Advanced Robotics

**DOI:** 10.3390/s21196340

**Published:** 2021-09-23

**Authors:** Ziqi Huang, Yang Shen, Jiayi Li, Marcel Fey, Christian Brecher

**Affiliations:** 1 Laboratory for Machine Tools and Production Engineering (WZL), RWTH Aachen University, D-52074 Aachen, Germany; m.fey@wzl.rwth-aachen.de (M.F.); c.brecher@wzl.rwth-aachen.de (C.B.); 2 UBTECH North America Research and Development Center, Pasadena, CA 91101-4858, USA; 3 Department of Statistics, University of California Los Angeles, Los Angeles, CA 90095-1554, USA; jiayi.li@g.ucla.edu

**Keywords:** artificial intelligence, machine learning, deep learning, digital twin, digital shadow, Industry 4.0, sustainability, sustainable smart manufacturing, robotics, review

## Abstract

Digital twin (DT) and artificial intelligence (AI) technologies have grown rapidly in recent years and are considered by both academia and industry to be key enablers for Industry 4.0. As a digital replica of a physical entity, the basis of DT is the infrastructure and data, the core is the algorithm and model, and the application is the software and service. The grounding of DT and AI in industrial sectors is even more dependent on the systematic and in-depth integration of domain-specific expertise. This survey comprehensively reviews over 300 manuscripts on AI-driven DT technologies of Industry 4.0 used over the past five years and summarizes their general developments and the current state of AI-integration in the fields of smart manufacturing and advanced robotics. These cover conventional sophisticated metal machining and industrial automation as well as emerging techniques, such as 3D printing and human–robot interaction/cooperation. Furthermore, advantages of AI-driven DTs in the context of sustainable development are elaborated. Practical challenges and development prospects of AI-driven DTs are discussed with a respective focus on different levels. A route for AI-integration in multiscale/fidelity DTs with multiscale/fidelity data sources in Industry 4.0 is outlined.

## 1. Introduction

Industry 4.0 and smart manufacturing are crucial fundamentals of modern industry and the national economy. Industry 4.0 aims to construct a universal networked architecture that addresses the interoperability and compatibility issues within and across all levels of the automation systems and factories, thus improving the flexibility and agility of conventional manufacturing. Equally indispensable to smart manufacturing is advanced robotics, which serves as an intelligent agent appearing in every corner of production lines. With the profound research and development of Industry 4.0 and artificial intelligence (AI), digital twin (DT) has drawn growing research attention [1,2,3,4]. As a digital replica of a physical entity, the *basis* of DT is the infrastructure and data, the *core* is the algorithm and model, and the *application* is the software and service. In recent years, the progressive aggravation of environmental problems, such as carbon emission and nuclear pollution, has required national industries to shift from conventional extensive economic growth to sustainable development. Achieving holistic sustainability commonly requires a balance within the *financial*, *environmental*, *social* and *governance* dimensions, i.e., *FESG* factors [5]. This increases the costs of manufacturing enterprises and simultaneously raises severe challenges for their organizations and processes. Against this backdrop, AI-powered DT technology is expected to adapt the traditional model-based approaches to the evolving boundary conditions and provide a demand-oriented, real-time capable evaluation basis to efficiently support decision making in multi-objective problems. There is already much research that discusses and characterizes DT from the view of general concepts and technologies [1,6,7,8,9,10,11] as well as certain fields, without targeted focus on AI, this novel enabler, i.e., product design [12,13], modeling and simulation [14], fault diagnostics and prognostics [15]. Practically, different engineering application scenarios have separate challenges and concerns. The grounding of DT and AI is even more dependent on the systematic and in-depth integration of domain-specific expertise. Currently, there is still a lack of comprehensive industry-oriented review of “AI + DT” technologies in the context of sustainability and circular economy. To further contribute to developing and landing of these general-purpose technologies (GPT) in smart manufacturing and advanced robotics, the following research questions (RQ) are proposed in conducting this survey:

RQ1: What are the current research and concrete case solutions on DTs?

RQ2: What is the current state of AI integration in DTs in the above two areas?

RQ3: What are the benefits of AI-enabled DTs, considering sustainability?

RQ4: What are the challenges in practice and future work with AI-enabled DTs?

This paper endeavors to address this research gap by revisiting current developments on DTs from a domain-specific perspective, analyzing implemented AI methods in each subarea, sorting out the role they play in sustainable development, and summarizing practical challenges in various application fields. The following contributions are delivered in this study:

1. The general development and application cases with common AI methods in AI-driven DTs of Industry 4.0 are concluded.

2. The advantages of AI-driven DTs in sustainable development are elaborated regarding the *FESG* factors, which enable a quantitative assessment of sustainability.

3. Challenges and development prospects of AI-driven DTs in smart manufacturing and advanced robotics are discussed with a respective focus on different levels.

4. A route for AI-integration in multiscale/fidelity DTs with multiscale/fidelity data sources along the product lifecycle is outlined.

### 1.1. Topic Definitions

#### 1.1.1. Digital Twin

The concept of DT was described by the National Aeronautics and Space Administration (NASA) as a multiphysics, multiscale, probabilistic simulation that uses physical models, sensor updates, fleet history, etc., to mirror the life of its twin [16]. Later, DT was indicated by Grieves and Vickers as a dynamic model based on massive amounts of information and computing capability that change over the lifecycle, including creation, production, operations and disposal [17]. Based on the architecture in [18], Tao et al. proposed an extended five-dimension DT model, comprising a physical entity, a virtual entity, service, data and connection [15].

#### 1.1.2. Digital Shadow

The concept “digital shadow” was mainly advocated by the German Academic Society for Production Engineering (WGP), whereby it is understood to be the sufficiently accurate representation of the processes in production, development and adjacent areas with the aim of generating a real-time capable evaluation basis of all relevant data [19]. Compared with the DT, a digital shadow does not require a high-resolution database, but a complete one [20]. Together with all of its subsystems, the digital shadow is designed as an information system or a multi-perspective information model to allow a more efficient operation of value creation systems, and is thus considered an enabler for data analytics in product lifecycle management (PLM) [21,22].

#### 1.1.3. Digital Thread

The concept of “digital thread” was initially proposed by the U.S. Air Force as a framework to merge detailed design models with model-based systems engineering (MBSE) conceptual and top-level architectural models [23]. This idea was further driven by the National Institute of Standards and Technology (NIST) with the purpose of exchanging information, including product design and quality and equipment performance and health, across the product lifecycle [24]. A single digital thread is, thus, created with the model-based ensemble of data in design, manufacturing, and inspection, which enables full-process traceability in a seamless, real-time, collaborative development among the project participants [25].

### 1.2. Topic Delimitation and Coverage

This survey provides in-depth insight into the current progress of AI-driven DT technologies, including the three aforementioned concepts, in Industry 4.0. Although there are various interpretations of the connotations and extensions of the DT, they share the same philosophy, namely, how to utilize digital replicas with near real-time capabilities to effectively enhance traditional organizations and processes across the product lifecycle, thereby improving industry competitiveness and optimizing resource allocation. Correspondingly, 5G communication and Internet of Things (IoT) technologies as well as standalone machine learning (ML) technologies without digital replicas are not the focus here. Over 300 manuscripts are covered on this basis.

### 1.3. Paper Organization

**Figure 1:**
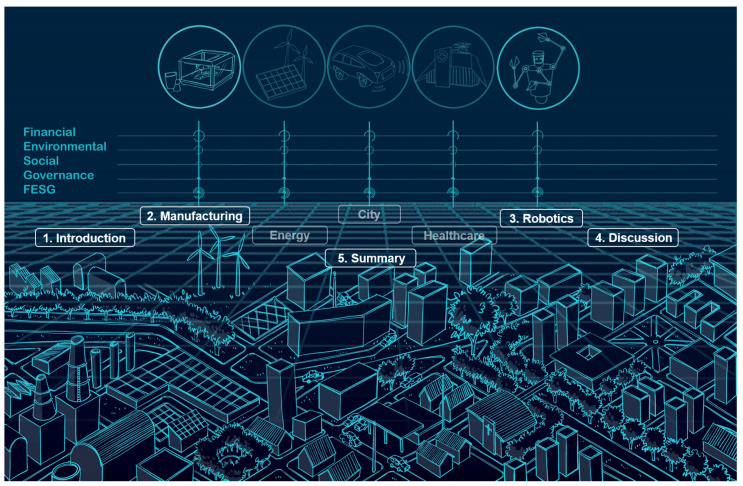
Overview of the review.

As illustrated in Figure 1, the rest of the review paper is organized as follows. In Section 2, we analyze the digital production twins toward sustainable resilient manufacturing at three different levels; in Section 3, we discuss the applications of DT in robots and human–robot interaction/human–robot collaboration. After dissecting AI-enabled case studies and branch-specific challenges of the development and deployment of DTs in industry verticals, Section 4 compares AI methods horizontally; Section 5 concludes the contributions of this paper and addresses the future work.

## 2. Sustainable Resilient Manufacturing

### 2.1. Overview

**Figure 2:**
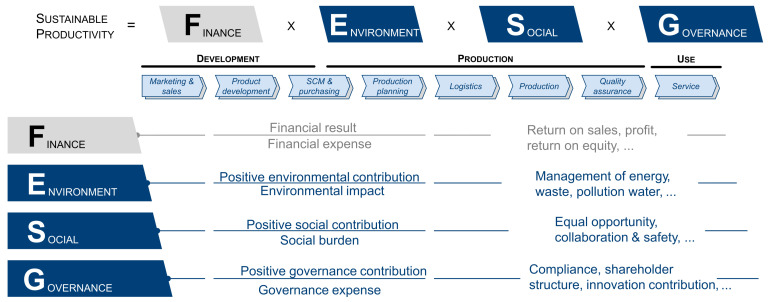
An illustration of holistic assessment of sustainable production [33]. © WZL—RWTH Aachen.

The core task of manufacturing industry lies in producing qualitative products in a productive and available manner. Balancing these partially competing objectives under time-varying boundary conditions is becoming a challenge in the course of ever-shortened decision-making horizons [26]. In recent years, intensive research has been conducted in the areas of Industry 4.0 [27], cyber–physical production systems [28,29], and integrative production techniques [30] to address the VUCA (volatile, uncertain, complex, ambiguous) market environment. The widespread application of simulation models and ubiquitous connectivity provide a solid foundation for building digital production twin throughout the product lifecycle, which is considered a key enabler for future manufacturing transformation and upgrading in the era of big data [1,31,32]. In the context of the circular economy and sustainability pledges (e.g., European Green Deal), traditional resource-intensive productivity thinking is being replaced by a future vision of a more ecologically minded society, namely “sustainable productivity” [33]. This understanding of productivity incorporates *FESG* factors as a novel indicator for quantitatively assessing sustainable production as well as the performance (with tangible and intangible services as well as business models) and value creation systems (consisting of resource, process, and organization) of manufacturing companies, thus pushing them to embrace the required sustainability transformation, as illustrated in Figure 2. Various research of DTs, including general developments and AI-integrated cases, in terms of enhancing the trilemma of productivity, availability, and quality (*financial*) toward sustainable resilient manufacturing (*environmental*, *social*, *governance*) are discussed in the following three levels: factory and shop floor (Section 2.2), machinery and equipment (Section 2.3), as well as process and material (Section 2.4).

### 2.2. Factory and Shop Floor

#### 2.2.1. General Developments

The megatrend toward the volatile market environment and individual customer demand poses new challenges, primarily for production systems and management in industrial enterprises, which can significantly influence the profitability and productivity of manufacturing. Within the various presented concepts and frameworks [34,35,36,37,38,39], automated production systems, including mixed reality assistance systems [40,41], could be rapidly modularized [42] and reconfigured [43,44,45], enhanced with AI [46,47] and sensors [48,49] and, in combination with cloud and edge computing [50], transformed into distributed control systems, while detailed production environments can be generated and updated in the form of 3D point clouds [51,52,53,54,55,56]. Based on these infrastructures, DT demonstrates the capability of handling increasingly complex operational problems, such as production planning and scheduling [57,58,59,60], production monitoring and control [61,62,63,64,65,66], quality control and management [67,68,69,70,71,72,73,74,75], as well as logistics [76,77,78], supply chain management (SCM) [79,80,81], disassembly and remanufacturing [82,83,84].

#### 2.2.2. AI-Integration

**Figure 3:**
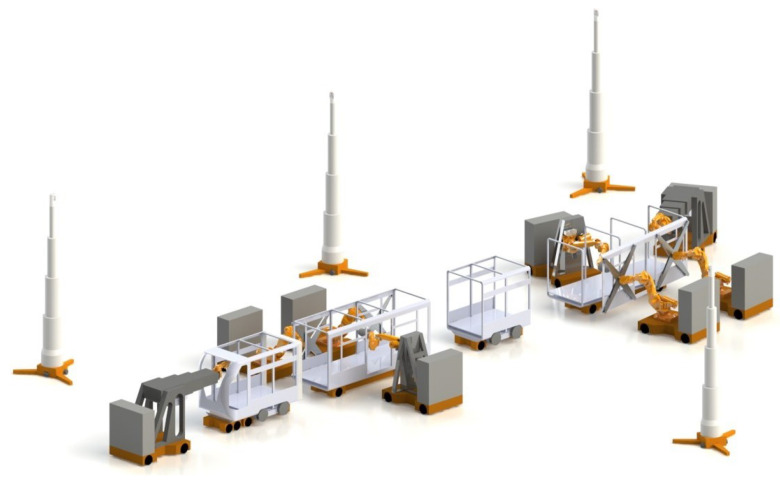
An illustration of DT in manufacturing: line-less mobile assembly system [85]. © WZL—RWTH Aachen.

The availability of industrial production data in a networked system landscape acts in this background as a technical enabler to increase the relevance of topics, such as AI and data-driven approaches. This further opens up new potentials for optimizing (novel) manufacturing systems, e.g., line-less mobile assembly systems in Figure 3, which enable agile assembly of large components by leveraging modeling and scheduling systems [85]. The primary objective of utilizing AI at this level is to improve the adaptability of DTs to dynamically changing boundary conditions on the factory and shop floor scale. Typical application subfields with AI-integrated DTs are *production planning* (Section 2.2.2.1), *production control* (Section 2.2.2.2), and *quality control* (Section 2.2.2.3), as shown in Table 1.

##### 2.2.2.1. Production Planning

According to the maturity model of production planning and control (PPC) proposed by Busch et al. [107], toward digitally connected, intelligent and adaptive PPC systems, AI is envisioned to support production planners in determining plans with improved key performance indicators (KPI), derive optimization measures and autonomously implement the identified measures prospectively to achieve better sequencing and reallocation of resources (*E*-factor). At the *green design* and *production planning* stage, decision tree could be applied in DT to create classic rules used in smart systems, thus facilitating decision making in multidimensional processes and strategic planning [86]. Hu et al. introduced a Petri-net-based dynamic scheduling approach via a deep Q-network (DQN) with graph convolution network (GCN) to solve the dynamic scheduling problems involving shared resources, route flexibility, and stochastic arrivals of raw products [87]. Metaheuristic methods, such as the genetic algorithm (GA) and other optimization methods [88,89,90,91] were similarly widely employed to deal with the scheduling problems in production lines.

##### 2.2.2.2. Production Control

At the *production control* stage, DNN [92], decision tree [93,94], and tree-based ensemble models, such as AdaBoost [93,94] and XGBoost [94], were implemented in assorted digital production twins to optimize resource allocation and manufacturing performance indicators in a timely manner (*E*-factor). However, the multi-objective problems at the factory level are usually interpreted as non-deterministic, polynomial-time hard, due to the complexity and dynamics in production environments. To address this challenge, reinforcement learning (RL), such as DQN and deep RL, were employed as a substitute for heuristic optimization and supervised approaches in various investigations, where the major task is normally mathematically formalized as a Markov decision process (MDP), with the objective of autonomously achieving the global optimal economic and logistic KPIs in a factory or logistic simulation environment [96,97,98,99] (*EG*-factor). In order to incorporate humans as a critical element in smart manufacturing, May et al. presented a concept for the situational selection of production control agents by forecasting human behavior modeled through a reinforcement learner [100] (*ES*-factor).

##### 2.2.2.3. Quality Control

At the *quality control* stage, classical supervised ML models, such as ANN, decision tree, and SVM, were expected to detect or predict potential deformations and surface deviations in production [101,102]. Deep learning (DL) computer vision models, including residual and convolutional neural networks were deployed to recognize eventual quality issues during the automatic production and machining features of parts [103,104], which could be further utilized to enhance the quality and efficiency of assembly processes [108] (*E*-factor), or retraced to the production planning stage in order to support decision making on the basis of historical production knowledge [109], as a “smart expert” in a collaborative environment (*SG*-factor). Following the general concept of integrating ML methods into the digital production twins [110], DTs of production systems in combination with MBSE can be modeled and adapted modularly as a virtual testbed, which in turn could provide a runtime environment for simulation-based optimization [111,112].

#### 2.2.3. Interim Summary

Business profitability remains the prerequisite for sustainable operation. DTs at this level elevate the productivity, resilience, and transparency of production processes, which enable end-to-end availability of data along the entire value chain as well as a holistic sustainability assessment on this basis. From a business development perspective, AI-enabled DT can additionally be considered a service agent [113], providing innovative smart services via DT network platforms [114] and subscription business models [115], thus contributing sustainably to long-term innovation for manufacturers, and helping them in accomplishing a paradigm shift from the one-time provision of production hardware to the ongoing delivery of manufacturing solutions (*SG*-factor). The importance of a diversified product and service portfolio is particularly evident in times of global crisis. For small- and medium-sized enterprises (SMEs), innovative services from research institutes and major manufacturing equipment suppliers can facilitate the conduction of the comprehensive balance sheet assessment covering FESG factors in order to achieve a smooth transition to sustainability.

### 2.3. Machinery and Equipment

#### 2.3.1. General Developments

The availability of production machinery and equipment has a direct impact on the efficiency of manufacturing processes and overall equipment effectiveness, and is, therefore, equally significant for sustainable production, especially regarding energy consumption and resource utilization (*E*-factor). Since their degradation and damage level can be affected by working operations as well as other disturbances in harsh manufacturing environments, such as lubricants, soiling, and temperature, the results from traditional wear and fatigue models are connected with considerable uncertainties. DT-driven condition monitoring (CM) and predictive maintenance (PdM) highlight the possibility for the process-parallel monitoring and diagnosis of the health status of the critical components (i.e., tools [116,117], bearings [118], ball screws [119], gears [15,120,121], pumps [122]) and the energy efficiency of the equipment [123,124] in order to handle the conflict between the unplanned maintenance operations and the resulting costs and productivity, particularly in SMEs, due to their limited capacity for the full deployment of a PdM strategy [125]. Additionally, DT-based optimal control [126,127] as well as machine dynamics issues [128,129] from a rotating system [130] to feed drive [131,132] are other important aspects.

#### 2.3.2. AI-Integration

Conventional model-based approaches of CM and PdM lie in the evaluation of process indicators, which are either recorded from sensors directly or determined indirectly by them. While the installation of external sensors (e.g., force measurement platforms and rotating dynamometers) increases costs and, more seriously, could negatively affect machine properties and manufacturing stability, DT combined with ML as a promising (soft) sensing technique provides an economically reasonable and sufficiently accurate approach to identifying such indirectly measurable process parameters (*EG*-factor). Table 2 provides an overview of AI-enabled cases in *condition monitoring* (Section 2.3.2.1), *predictive maintenance* (Section 2.3.2.2), and *dynamics and control* (Section 2.3.2.3).

##### 2.3.2.1. Condition Monitoring

For *condition monitoring* of machining tools and processes, Königs et al. [133] proposed a hybrid modeling method, whereby an ANN utilized machine internal signals and the cutter-workpiece engagement map generated from a real-time virtual machining simulation as inputs to predict the cutting force as an essential indicator. Su et al. proposed another machining force prediction model based on the cutter frame image data using CNN [134]. Gao et al. [135] introduced a deep lifelong learning method based on CNN and an SVDD-based (support vector data descriptor) detector, which enables recognizing multiple tool defects (crazing, patches, scratches, etc.) with novel classes by learning relevant tool images. CNN-DLSTM based transfer learning [136], generative models [137], and dictionary learning [138] were furthermore employed to assist in abnormal signals detection and fault prognosis (*E*-factor).

##### 2.3.2.2. Predictive Maintenance

Based on the monitoring status, health indicators, such as remaining useful life (RUL), could be estimated, particularly under non-stationary operation conditions, thus enabling effective *predictive maintenance* planning (*E*-factor). Besides statistical [140,141] and hybrid modeling [142] approaches, DL models for time-series forecasting, such as LSTM [144,145,146], were adopted in numerous studies to estimate wear status and equipment utilization. However, the acquisition of massive, structured and labeled data, especially regarding complex rotating equipment and components, is normally tied up with high costs since the fault-free operation is a frequent case in production. Considering this fact, several attempts with unsupervised and semi-supervised learning models, e.g., GMM (Gaussian mixture model) [147], SSAE (stacked sparse autoencoder) [148,149], and GAN (generative adversarial network) [150], were investigated in order to reduce the reliance on historical failure data in terms of prognostics and health management (PHM). Regarding the cost factor, Palau et al. [152] provided a methodology to assess the optimal multi-agent system (MAS) architecture for collaborative PdM in large fleets of industrial assets by using a distributed k-means clustering algorithm (*ES*-factor).

##### 2.3.2.3. Dynamics and Control

The manufacturing stability and reliability are furthermore closely related to the dynamic behavior of production machines and process machine interactions (e.g., chatter), namely, *dynamics and control*, which are due to complex damping effects and structure modification (e.g., tool changing), which are, in practice, difficult to estimate accurately and/or efficiently (*E*-factor). In this respect, Kabaldin et al. [154] selected RNN to estimate the statistical model of the dynamic state in cutting. ML models, such as ANN [155], and probabilistic modeling methods, such as the Gaussian process [156,157,159], could likewise be adopted to develop a surrogate model and implemented in a control context [157,158,160]. Alternatively, model order reduction techniques can transfer highly detailed and complex simulation models to other domain and life cycle phase, e.g., building efficient finite element model for dynamic structural analysis through reducing the degree of freedom, while maintaining required accuracies and predictability [161,162,163].

#### 2.3.3. Interim Summary

**Figure 4:**
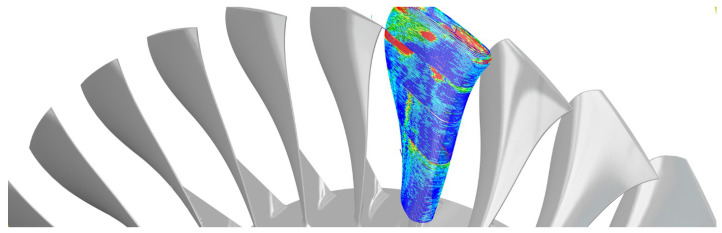
An illustration of DT in manufacturing: CM in the networked, adaptive production [165]. © Fraunhofer IPT.

As the backbone of the manufacturing industry, research on machinery and equipment has a protracted history, yet complex and varying working conditions with relatively sparse datasets in practice frequently make it challenging to transfer research findings, commonly in the form of elaborate analytical/empirical models, to real industrial environments. From a data perspective, the networked production landscape provides an additional unique opportunity to use each manufacturing system as a “test bench” in order to continuously enhance the database and the amount of labeled training samples regarding these indirectly measurable and non-measurable indicators. The improved availability of scarce datasets prospectively extends the previous model boundaries and transferability through ML/DL in equipment fault diagnosis and system behavior prediction, which remain as central concerns within the scope of sustainable manufacturing (*E*-factor). High data rates empowered by 5G technology [164] similarly open up novel prospects for research on AI-driven DTs in the field of PHM, e.g., for sensing high-frequency phenomena in high speed/performance cutting. Figure 4 illustrates the application of digital twin for CM and PdM in the networked, adaptive production.

### 2.4. Process and Material

#### 2.4.1. General Developments

At this level, we are more concerned about the quality issues and mechanical properties of manufactured parts. The quality of parts is normally determined in quality assurance after the entire machining process by testing quality specifications, such as form and position tolerances, as well as surface roughness. Regarding the optimization of the current quality control loop and the associated costs, the digital process twin represents a core element in modern manufacturing. For instance, the surface quality of parts can be estimated in parallel to the process within a GPU-enabled (graphical processing unit) material removal simulation with a subsequent virtual measurement, which significantly reduces the latency between machining and the detection of defective parts [166,167]. A sufficiently accurate virtual representation of the machining process [26,168,169,170,171] also enables cause-and-effect analysis and, therefore, robust process design and control [172,173,174,175,176] as well as the exploitation of potential process productivity [177]. Further cases of production processes with knowledge-based approaches include welding [178,179], injection molding [180,181], linear winding [182], tape laying [183], metal forming [184], laser polishing [185], automated fiber placement [186], sheet molding compound [187], fused filament fabrication [188], and metal additive manufacturing (AM) [189,190,191,192,193].

#### 2.4.2. AI Integration

While the refining scale of models allows deeper insights into the mechanism of manufacturing processes, their modeling takes correspondingly more time. In addition to leveraging the parallel computing power of GPUs for near real-time process simulation, it is common practice in engineering to compensate online with the results of preprocessed offline simulations. ML and DL methods can thus be trained as surrogate models in order to efficiently update time-consuming numerical simulations at the process and material scale. These ML/DL-equipped lightweight models could be used for faster production ramp-up as well as soft sensory for inline-quality monitoring (*EG*-factor). They also deliver additional process understanding, thus optimizing the space-time yield and accelerating process development [194]. Table 3 presents a summary of cases of AI-driven digital process twins for major manufacturing techniques involving *metal cutting* (Section 2.4.2.1), *metal AM and laser material processing* (Section 2.4.2.2), and *composite material processing* (Section 2.4.2.3).

##### 2.4.2.1. Metal Cutting

For conventional *metal cutting* techniques, e.g., milling and drilling, the part quality is primarily determined by sophisticated machine behavior and cutter-workpiece engagement, which can become particularly intricate in multi-axis machining or for parts with thin-walled structures. Zhao et al. constructed a self-learning surface roughness prediction model based on pigeon-inspired optimization and SVM in order to stabilize the part quality with a self-adaptation adjustment method [195]. Approaches combining ANN and semantic modeling for fatigue and quality prediction were discussed in [196,197,198]. Following the philosophy of DfX (Design for X), Zhou et al. utilized the DDPG (deep deterministic policy gradient) approach to optimize decision making, according to the performance and machinability of parts, which could shorten cycles and save costs in the product development [199] (*SG*-factor). At the material scale, evolutionary algorithms, such as PSO, were investigated by Hardt et al. to inversely identify the material model parameters in finite element (FE) simulations of orthogonal cutting processes [200].

##### 2.4.2.2. Metal AM and Laser Material Processing

For advanced *metal AM and laser material processing* techniques, e.g., laser powder bed fusion (LPBF) and laser melting deposition (LMD), research efforts focus on the subtle impacts of thermal effects on materials, such as the microstructure and parts’ distortion, during such non-contact processes. As Gaikward et al. in [201] presented the temperature distribution of parts, predicted based on a graph–theoretical computational heat transfer approach and subsequently combined it with an SVM model in order to detect potential quality faults in printing processes. Another attempt of grey box modeling for build quality in dependency of process parameters and in situ sensor signatures was proposed in [202] by Gaikward et al., where the a priori knowledge of physical processes was incorporated into three sequentially connected shallow ANNs and consequently achieved better performance in comparison with purely data-driven methods (CNN, LSTM, RNN, among others). With respect to DfX and knowledge engineering, Ko et al. employed CART to predict additive manufacturability, which was further fed back to a knowledge-query formulation phase in order to continuously construct and broaden an AM knowledge base [203] (*EG*-factor). In addition, HMM and k-means demonstrated their applications for quality assessing and monitoring in [204,205].

##### 2.4.2.3. Composite Material Processing

For similarly novel but not yet matured *composite material processing* techniques, e.g., lightweight production of fiber-reinforced polymers, hybrid modeling of non-measurable process variables and process signatures refined therefrom, are anticipated to provide more process understanding and transparency (*EG*-factor). Stieber et al. proposed a CNN-based transfer learning approach to in situ monitor the polymerization progress of resin transfer molding (RTM) [206]. Hürkamp et al. implemented AdaBoost, XGBoost, and random forest as finite element surrogate models to predict temperature distribution during the fabrication of overmolded thermoplastic composites [207]. Similarly, DNN was applied by Pfrommer et al. as surrogate modeling to optimize the manufacturing process parameters of a composite textile draping process [208]. A probabilistic ML approach with statistical inference was developed by Ghanem et al. to efficiently update numeric simulations of a composite material system and reveal multi-scaling relationships of mechanical properties and behaviors [209]. Kanyuck et al. introduced a methodology for identifying sheet material parameters using the ISRES algorithm and implemented a thin-shell simulator for predicting the material behavior, which enabled a defect-free layup of prepreg composite sheets in human–robot collaborative cells [210] (*ES*-factor).

#### 2.4.3. Interim Summary

**Figure 5:**
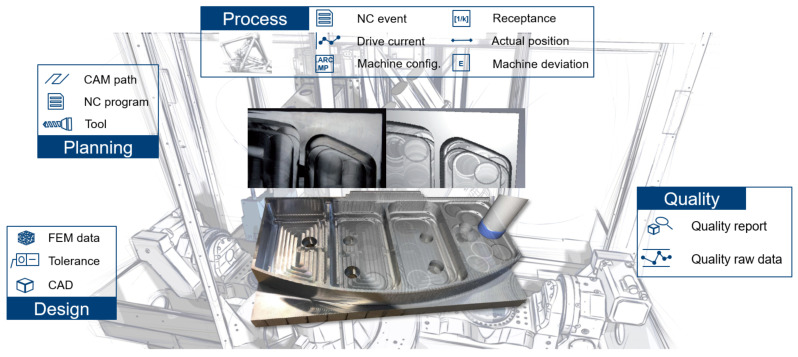
An illustration of DT in manufacturing: workpiece quality monitor [26]. © WZL—RWTH Aachen.

AI-driven digital process twins are envisioned to learn and interpret implicit correlations between manufacturing processes and material/process/environmental parameters from an aggregation of (heterogeneous) data with the objective of optimizing process development, production ramp-up, and quality assurance cycle. From an engineering implementation perspective, we have noted that despite the significance of algorithms and models, novel sensor technologies [167,213,214,215,216,217] and networked digital process chains [218,219,220,221,222] should not be neglected, as they are essential pillars for constructing DTs and can considerably influence the effectiveness and efficiency of their development and deployment in practice. Figure 5 shows an example of a DT dynamically mapping the manufacturing process of an aerospace part and the data sources involved from the contextualized CAD-CAM-CNC-CAQ process chain. As yet, these immature manufacturing techniques, such as 3D printing and lightweight production of metals and composites, are still set up in a trial-and-error manner [223]. Their maturation will not only lead to innovative concepts (*SG*-factor) and resource savings (*E*-factor) in the design and manufacturing phase, but also provides a pivotal basis for the sustainable operation, maintenance, and reuse of the product downstream, thus reducing energy consumption and carbon emissions across the product lifecycle. The resulting sustainability upgrades are, therefore, all-encompassing.

### 2.5. Challenges and Outlook

Key practical challenges and future work are included as follows.

**Figure 6:**
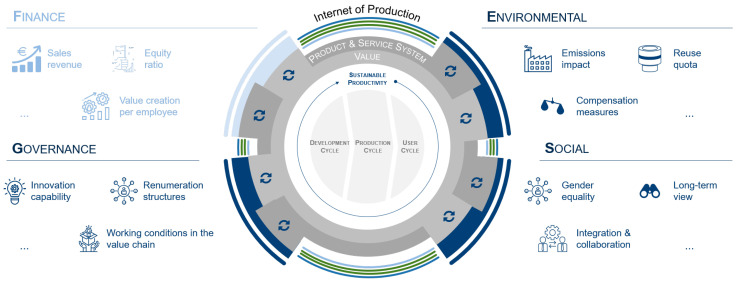
An illustration of sustainable resilient manufacturing [33]. © WZL—RWTH Aachen.

1. Vertical interoperability in the production context (*basis/infrastructure*): despite various proposed concepts and cases, a standard framework for developing and deploying multi-scale DTs combining with AI methods has not yet been established. A valid reference framework covering separate manufacturing levels should be defined in the future. For this purpose, interdisciplinary cooperation is indispensable [224].

2. Horizontal interoperability in the production context (*basis/data*): sufficiently high data quality is a fundamental prerequisite for building AI-driven DTs. In the production context, however, the usage of heterogeneous data sources from different software and hardware across the product life cycle is associated with high costs. Through consistently and comprehensively contextualizing and interconnecting of these data silos, the full potential of DTs can be exploited in terms of further optimizing the agility, traceability, resiliency, and transparency of current manufacturing. Interfaces for standard information exchange are, therefore, imperative [225].

3. High-fidelity, lightweight models with uncertainty quantification (*core*): real manufacturing processes are characterized by numerous, partly stochastic variables with highly complicated cause–effect relationships. In the course of increasing product variants driven by the market, frequently changing technical boundary conditions bring more parameterization efforts and potential uncertainties. Conventional, complex and non-real-time capable models are, therefore, gradually reaching their limits in the industrial environment. In this sense, data-driven approaches with the incorporation of prior knowledge promise to extend the current model boundaries, thereby improving the industrial objectives in terms of productivity, availability, and quality (*F*-factor).

4. Smart services and business models (*application*): novel digital platforms contribute to long-range collaboration and innovation, ensembling isolated AI-equipped DT-solutions from diverse levels, and thus offering new opportunities for manufacturers to deliver products and solutions sustainably, e.g., through the X-as-a-Service (XaaS) model. Moreover, the transparency distilled from the entire product value chain serves as an on-demand and real-time capable analytical foundation for the holistic assessment of sustainable resilient manufacturing in order to achieve a balance in the *financial*, *environmental*, *social*, and *governance* dimensions. The concept of “sustainable productivity” based thereon is depicted in Figure 6 selected from the white paper by Boos [33]. Therein, the vision of “Internet of Production” provides the infrastructure for harnessing data along the product lifecycle [226], while the transparency generated from the (AI-powered) DTs enables product design, manufacturing, and usage exclusively, according to actual demand-, quantity-, and user-oriented requirements [33].

## 3. Advanced Robotics

### 3.1. Overview

**Figure 7:**
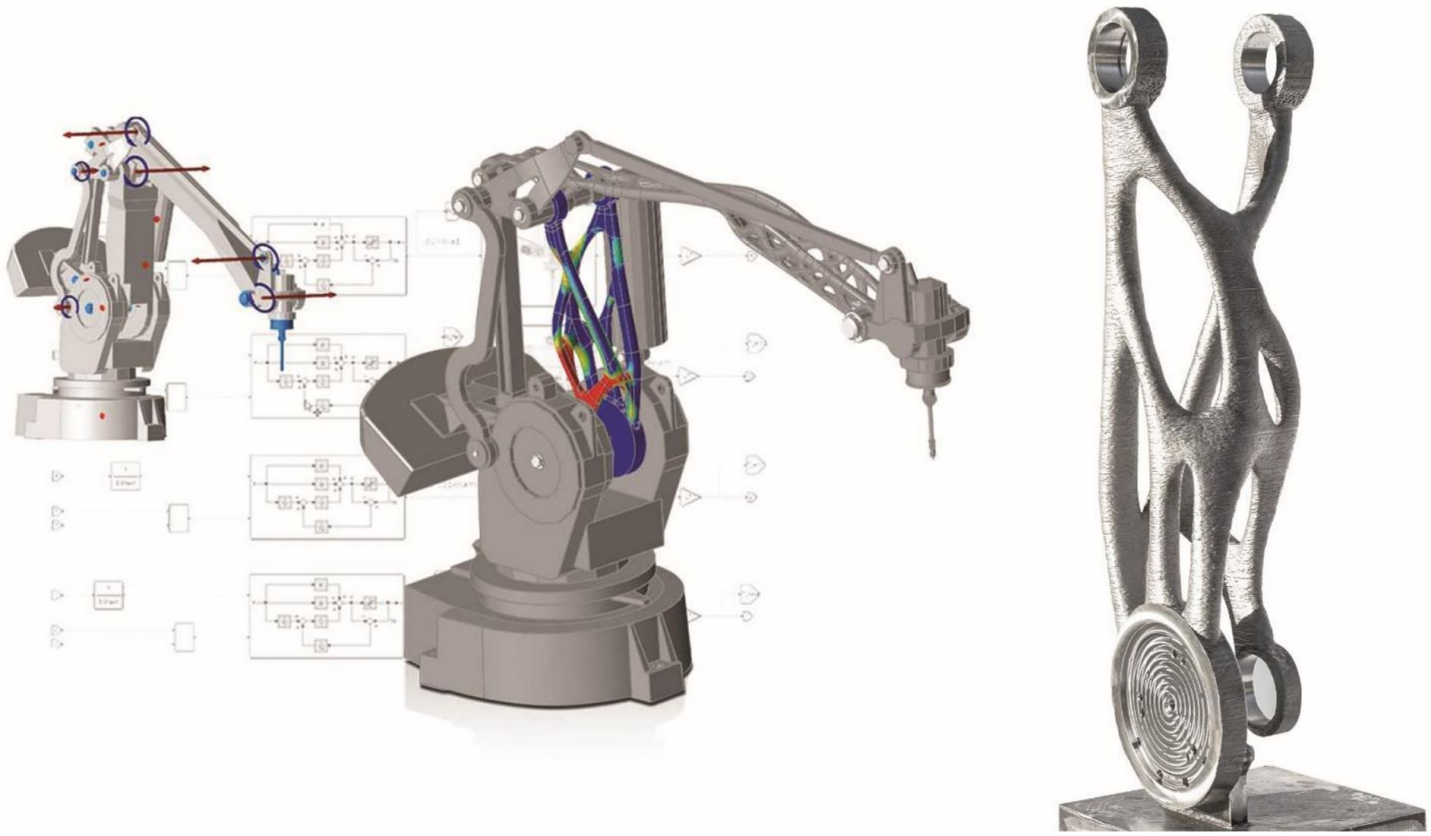
An illustration of DT in robotics: in a joint project MX3D, ABB, and Altair demonstrated how a 3D-printed robot can be improved by using a digital twin process to achieve more precise positioning [259]. ©Altair.

**Figure 8:**
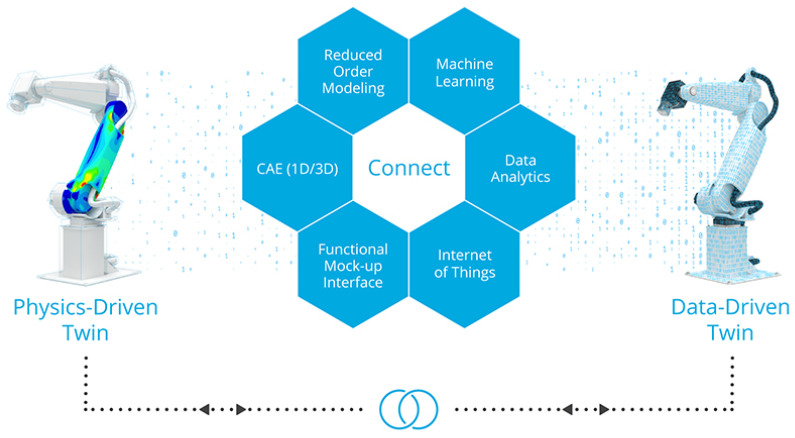
An illustration of DT in robotics: Altair digital twin platform [288]. ©Altair.

Along with the widespread deployment of robotic systems in industry and daily life [227], having a digital twin of robots becomes more and more critical in practical scenarios, such as multi-robot coordination/collaboration as well as those that require safe human–robot interaction (HRI) and/or complex human–robot collaboration (HRC) [228,229,230,231], which place human safety as a high priority, thus helping to create a sustainable working environment (*SG*-factor). Many have used traditional simulation/cloud framework to attempt robot DT implementations, such as the ones shown in Figure 7 and Figure 8. Other examples could be found in kinematics [232], communication [233,234], control [235,236,237], planning [238], and industrial robot energy modeling [239], in use cases like welding [240], cleaning [241], pick-and-place [242], assembly [243,244,245,246], manufacturing [247], warehouse [248,249], maintenance [250], and construction [251]. Some well-known robotics simulation tools are Gazebo [252], MuJoCo [253], and CoppeliaSim (aka V-REP) [254]. Recently, new concepts and cases utilizing artificial intelligence towards semi- and fully autonomous robotic systems have been reported, e.g., transfer learning [255] and imitation learning (also known as apprenticeship learning or learning from demonstration) [256,257]. While traditional DTs have been developed for systems that we have a solid grasp of (in other words, *model-based*), data-driven and AI-equipped DTs help with complex robotic systems for which building high-fidelity dynamics models is not feasible (*model-free*). The latter has been applied in more and more cases, even for biomimetic robotic system development (e.g., robotic fish [258]). Table 4 summarizes AI-equipped DTs and categorizes them based on learning algorithms used and subfields, such as control (detailed in Section 3.2), planning (detailed in Section 3.3), and HRI/HRC (detailed in Section 3.4).

### 3.2. Control

A key part of modern robotic control is feedback, which expects accurate information collected from physical sensors installed on robots and in the external environment and to provide commands for the next loop of execution. Sometimes, real time is required to enable safer controllers on these robotic systems. Many efforts utilizing AI + DT have been attempted in this subfield. Compared with traditional DTs, those equipped with AI and driven by data have advantages of gaining better generalizability and higher adaptability in a varying environment, and accomplishing nontrivial sensing/manipulation tasks. At the *sensing* stage, Jin et al. reported a smart soft-robotic gripper system based on triboelectric nanogenerator sensors to capture the continuous motion and tactile information for soft gripper control, where PCA and SVM were used to realize real-time prediction [260]. Data- or AI-driven approaches can also be found in other touch/haptic/force sensing for obtaining better system understanding and task performance [280,281,282]. One level higher, at the *controller* stage, Verner et al. implemented online reinforcement learning via a fabricated digital twin, to enable a humanoid robot to lift a weight of unknown mass through autonomous trial-and-error search [261]. Similarly, in [283], Grinshpun et al. reported the development and deployment of control algorithms for soft robots, with particular reference to industrial peg-in-hole insertion tasks. Vrabič et al. used DT and gradient descent to optimize controller parameters of a mobile robot [262]. More data/AI-driven examples in robot control include [284,285]. At the *application* level, one example is that Oyekan et al. utilized vision-based Markovian chain to automate fan-blade reconditioning for aerospace maintenance, repair and overhaul with a 6DOF robotic arm [263] (*E*-factor). Another example is that Klamt et al. built a DT for the famous CENTAURO robotic system to support rescuers on disaster response missions [264] (*S*-factor).

### 3.3. Planning

Once the low-level robotic control is in good shape, the high-level robotic planning, as another critical part of realizing autonomous robotic systems, comes into play. Unlike the low-level control subfield, which emphasizes the system response and robustness, high-level planning focuses more on strategically finding a close-to-optimal solution among all feasible options, under specific constraints. Compared with traditional search-based motion planning algorithms, reinforcement learning has demonstrated huge potentials in bringing intelligence to complex systems planning, e.g., a humanoid robot with high degrees of freedom (DOFs) [286]. However, it is usually difficult to train reinforcement learning because obtaining the data from the real physical system is both finance- and time-consuming. The curse of dimensionality may also disable the system from learning something useful [287]. As a robotic system’s digital twin grows up and provides reliable data, the combination “DT + RL” seems to be a promising approach (*SG*-factor): in [265], Matulis et al. integrated digital twin and reinforcement learning for a 6DOF robotic manipulator to plan pick-and-place motions; in [266], Liu et al. proposed a multitasking-oriented robot arm motion planning scheme based on deep reinforcement learning and twin synchro-control; in [267], Xia et al. proposed a DT to train deep reinforcement learning agent for automating smart manufacturing systems; and in [268], Zhao et al. demonstrated collision avoidance for a number of UAVs in a confined airspace, using LSTM-MACG. While RL has become popular in recent years, it is not the only AI strategy that is integrated with DTs. For example, Bansal et al. developed an ant colony optimization algorithm for industrial robot programming in a digital twin [269].

### 3.4. HRI and HRC

Under the context of Industry 4.0, one of the very important benefits that DT could bring to robot-involved scenarios is safer human–robot interaction and human–robot collaboration [289,290] (*SG*-factor). HRI and HRC scenarios are, intuitively, more complex and challenging than robot-only applications, due to not only the uncertainties in the environment and sensors, but also the randomness and diversity of human behavior. One of the advantages of AI-equipped DTs over conventional ones is the ability to better respond to these (sometimes implicit, such as [291]) variables that are nontrivial to exhaustively model and analyze. For example, in [292], Wang et al. proposed a real-time process-level digital twin for collaborative human–robot construction work. The proposed DT utilized immersive virtual reality (VR) and combined the as-designed BIM model and the evolving as-built workspace geometry obtained from on-site sensors, to provide the capability for both planning and improvising. Similarly, in [270], Li et al. proposed DL-based human standing-posture recognition in HRC. Other supervised learning applications, such as visual question answering for the HMC system, are included in Table 4 [271,272,273]. A supervised/unsupervised learning example is [274], where Wang et al. used FFT–PCA–SVM–based DT for human–robot interactive welding and welder behavior analysis. In [275], Lv et al. proposed a reinforcement learning–based DT for improving medical equipment assembly efficiency during COVID-19.

### 3.5. Robot Maintenance and Other Applications

Robotic systems, like other machines with a physical entity, have downtime and need maintenance as well (*E*-factor). Without explicitly mentioning the concept of DT, Khalastchi et al. and Vallachira et al. reported applications of using data-driven methods in robot anomaly/failure detection in [293] and [294], respectively. In [276], Anton et al. used deep learning equipped DT for global system health monitoring as well as predictive, customized maintenance. Similarly, in [277], Aivaliotis et al. integrated degradation curves in the predictive maintenance of industrial robots. Some other applications, such as using the Monte Carlo learning method in calculating the workspace of a serial robot manipulator [278] and random forest based estimation of lawn grass lengths for robotic lawn mower [279], can be found in Table 4.

### 3.6. Challenges and Outlook

There are several key challenges in developing and implementing digital twins in the field of robotics [295,296]. First, the multibody physical simulation is intrinsically difficult, due to the complex interaction properties at the interfaces of robot–robot, robot–human, and robot–environment. In addition, since robot movement can often be extremely fast (e.g., in an assembly line), real-time feedback from sensors is critical to the digital twin’s effectiveness in making short-term decisions. Many researchers in academia choose to use simulators, such as Gazebo (e.g., [297,298]), as the simulation environment for developing robots, but even after years of evolution, those robotic simulators still have many unsolved limitations and may require high-performance computing (HPC) platforms. Second, inputs and disturbances from the human user add another layer of uncertainty and unpredictability to the whole collaborative system/workspace, and thus, compromise HRI/HRC safety. While standard-compliant (e.g., ISO 13482) safety measures must be facilitated on both physical and digital sides, having virtual reality or augmented reality (AR) technologies involved is another thread to make the human–robot interaction intuitive [299]. Rückert et al. also suggested the consolidation of product life cycle information within human–robot collaborative assembly tasks [300].

## 4. Discussion

DTs have shown remarkable potentials to contribute to industrial economic growth or *F*-factor (i.e., the productivity, availability, and quality of manufacturing) while continuously upgrading sustainable aspects, such as the *E*-factor (e.g., reduced carbon emission and resource consumption through CM, PdM, 3D printing and lightweight production of metals and polymers) and *SG*-factor (e.g., enhanced working conditions, collaboration and innovation through HRI/HRC, XaaS model, and intelligent/soft sensing of novel production indicators) as summarized in Table 5. In this range of cross-domain engineering cases, AI techniques arm digital twins with tools to create models based on observed behavior and historical data, which improves the efficiency of data analysis and increases prediction accuracy by integrating data from a collection of disparate and incompatible sources.

The AI techniques involved in digital twins can be roughly categorized into four classes: supervised learning, unsupervised learning, reinforcement learning and other intelligent computational methods. Supervised learning algorithms refer to machine learning methods in which models are trained using labels. Typical supervised learning methods used in digital twin include supper vector machine (SVM) [195,201], decision trees [86,93,94], k-nearest neighbors [102], convolutional neural networks (CNN) [103,135,202,206,270] and recurrent neural networks (RNN) [202]. In practice, data labeling can be an expensive task. Most supervised learning algorithms require a large amount of labeled data at the training stage to obtain a model with high prediction accuracy. In general, the more complex the architecture is, the more data are needed to produce viable results. The results of supervised learning algorithms also depend on the selection of feature vectors and the accuracy of labeling.

In unsupervised learning methods, there is no labeling of data required, and the model is expected to infer patterns from the unlabeled input data. Clustering algorithms, such as principle component analysis (PCA) [260,274] and k-means methods [205] and generative models using generative adversarial network (GAN) [137,150] and variational autoencoders (VAE) [150] all use unlabeled data at the training stage, thus falling into the category of unsupervised learning. One of the challenges in applying unsupervised learning methods is that the number of clusters is normally not known a priori. For clustering algorithms, the clusters are determined by the metric used to measure similarity—Euclidean, cosine, Gaussian distance—of which the criteria are not clear for a given task. Reinforcement learning algorithms are concerned with how intelligent agents ought to take actions in an environment in order to maximize the notion of cumulative reward. Researchers have applied reinforcement learning algorithms, including Q-learning [96], deep reinforcement learning [95,97,267] and deep deterministic policy gradient [199,266,275] to optimize the decision-making process of box sorting, conveyor systems and other DT scenarios. The performance of a reinforcement learning system generally heavily depends on the correctness of data logging and the choice of reward structures. Logging to incorrect references might corrupt the information and lead the whole system to break down during training.

**Figure 9:**
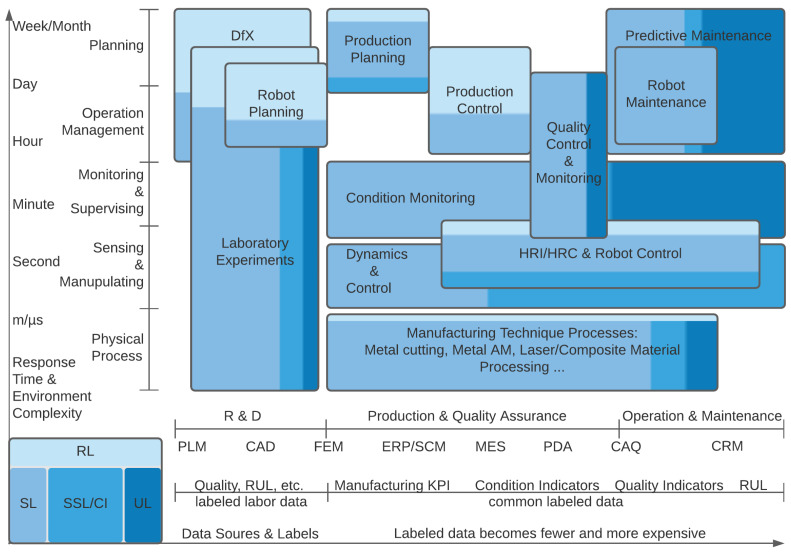
An illustration of AI-integration in multiscale/fidelity DTs along the product lifecycle.

From the above, the fidelity of AI-driven DTs depends largely on the model granularity, the selection of which (*core* level) is, in turn, related to the environment complexity (*application* level) and dataset quality (*basis* level). Based on the review of over 300 manuscripts, we sketched a route for AI-integration in multiscale/fidelity DTs in the real-world case of multiscale/fidelity data sources, as outlined in Figure 9. This multiscale nature is reflected in both spatial and temporal terms. Vertically, the demand for real-time capability in digital twins alters with different levels of the automation pyramid. While the higher-scale planning and management typically require a longer response time to deal with changing production environments, time-sensitive sensing and manipulation rely on the in-depth insight of physical processes gained through refining scale modeling. Horizontally, datasets of varying difficulty and fidelity are constructed from heterogeneous sources over the product lifecycle. In the development phase, simulation tools and laboratory experiments separately provide comparatively large amounts of data with a moderate degree of fidelity and limited high-accuracy data under controlled conditions. As products are manufactured and utilized, obtaining reliable labeled data, such as quality indicators and RUL, becomes increasingly scarce and expensive. On this basis, AI methods can be developed and deployed in pipelines. In general, supervised learning remains the most stable and extensively used approach in digital twins. Reinforcement learning is often advantageous in scenarios with complex environments and long response cycles, unsupervised and semi-supervised learning are quite proactive in state detection and lifetime prediction, and traditional intelligence methods are still commonly used and can be flexibly combined with other learning methods.

## 5. Summary

Traditional profit-maximizing industrial technologies have transformed human society while causing significant—and mostly irreversible—negative impacts on the environment and climate. Sustainable development strategies are gaining attention, but still have a long way to go, as all aspects within the *FESG* dimensions have to be balanced in real implementation. According to a report from the WBA Tooling Academy Aachen [301], classic evaluation for manufacturing companies with a focus on value added and equity ratio could show a significantly worse outcome in the extended analysis that incorporates *ESG* criteria in the balance sheet. For sustainable thinking and practices, which have been increasingly deliberated and advocated worldwide, digitalization and AI are powerful enablers. Through consistently linking, processing, and analyzing all available data across the entire value chain, and connecting them to a digital twin or shadow, a holistic assessment of sustainability, namely, the consideration of *ESG* factors alongside profitability (*F*-factor), can be achieved so that companies can develop and operate sustainably. For Industry 4.0, which is facing new challenges from climate-neutral products and production, AI-driven DTs are expected to provide the additional manufacturing transparency and understanding that enable a demand-oriented and real-time capable analytical foundation, considering *FESG* factors along the product lifecycle. The main contributions of this study are concluded as follows.

1. The general development and application cases with common AI methods in AI-driven DTs of Industry 4.0 are concluded.

2. The advantages of AI-driven DTs in sustainable development are elaborated regarding the *FESG* factors, which enable a quantitative assessment of sustainability.

3. Challenges and development prospects of AI-driven DTs in smart manufacturing and advanced robotics are discussed with a respective focus on different levels.

4. A route for AI integration in multiscale/fidelity DTs with multiscale/fidelity data sources along the product lifecycle is outlined.

In the past, typical production issues, such as predicting the behavior of machine tools as single systems, were already well approximated by complex analytical and empirical models; their application in industry environments, however, has often been handicapped, due to both the lack of real-time capabilities and transferability in varying frameworks. AI promises in this circumstance to extend the model boundaries of traditional model-based approaches, particularly within changing boundary conditions. As regards the relatively sparse and expensive datasets in engineering, incorporating prior knowledge can reduce the dependence of pure data-driven approaches on the amount of historical data and improve the predictivity and transferability of models. Moreover, statistically-based uncertainty quantification similarly plays an essential role in building AI-driven DTs, as it allows to assess the reliability of the modeled results and, thus, influences their acceptance and the decision-making based thereon in real, high-risk engineering.

Meanwhile, we have noted that the development and deployment of AI-enabled algorithms and models, the *core* of the DT, are still constrained by the current infrastructure and that constructing the latter requires interdisciplinary collaboration and integration of domain-specific expertise (*basis* level). New breakthroughs in novel sensors and benefits from 5G communications and OPC UA TSN are expected in the near future. In terms of the *application* level, while smart service and new business models facilitate the paradigm shift for manufacturers, a prerequisite is the willingness to share data and knowledge with partners to a healthy extent. Standardized concepts for data ownership and data security must constitute the basis for this. A further survey on the AI-driven DT technologies in the application fields of renewable energy, smart city and mobility, and healthcare will be covered in future work. By reorganizing and aggregating several highly relevant topics both horizontally and vertically, we believe that a synergistic effect will emerge that can allow the work in this study to contribute to more AI-driven, DT-related research and help various branches build new developments in their respective sustainable and smart areas.

## Figures and Tables

**Table 1:** Summary of AI-enabled DTs in smart manufacturing: **factory and shop floor level** (Section 2.2).

Subfield	AI-Category	Key Methods	Application-Case	Ref.
Production Planning (Section 2.2.2.1)	Supervised Learning	Decision tree	Material selection; tool holder selection; tool wear level prediction	[86]
	Reinforcement Learning	DQN	Dynamic scheduling of flexible manufacturing systems	[87]
	Computational Intelligence	GA, DES	Optimization of production scheduling	[88]
		GA	Optimization of smart assembly lines	[89]
		Supernetwork	Scheduling optimization of a gear production workshop	[90]
		Multi-objective optimization	Design of automatic flow-shop manufacturing system	[91]
Production Control (Section 2.2.2.2)	Supervised Learning	PGM	Resource allocation for sequential manufacturing operations	[36]
		DNN	Assembly commissioning process optimization	[92]
		AdaBoost, CART, Gradient boosting	Identification of real-time factors that influence throughput in a semiconductor fab	[93]
		AdaBoost, XGBoost, decision tree	Optimum yield of the light oil	[94]
	Reinforcement Learning	Deep reinforcement learning	Geometrical quality improvement in assembly	[95]
		Q-Learning	Box sorting in a material flow control system	[96]
		DQN	Optimization of the workflow in a semiconductor wafer processing plant	[97]
		DQN	Optimization of conveyor systems	[98]
		TRPO	Optimizing order dispatching	[99]
		TRPO	Human behavior forecasting	[100]
Quality Control (Section 2.2.2.3)	Supervised Learning	ANN	Welding quality prediction (deformation) in an assembly line	[101]
		Decision tree, k-NN, SVM	Anomaly detection of surface deviations of a truck component	[102]
		CNN	Feature recognition of parts	[103]
		ResNet	Recognizing machining features on CAD by inputting its views	[104]
		CNN, autoencoder, U-Net	Fringe projection profilometry for 3D reconstruction	[105]
Logistics	Computational Intelligence	Self-learning generic positioning	Abnormal condition detection and location information preservation	[106]

DQN: Deep Q-Network; GA: Genetic Algorithms; DES: Discrete Event Simulation; PGM: Probabilistic Graphical Model; DNN: Deep Neural Network; AdaBoost: Adaptive Boosting; CART: Classification and Regression Tree; XGBoost: eXtreme Gradient Boosting; TRPO: Trust Region Policy Optimization; ANN: Artificial Neural Network; CNN: Convolutional Neural Network; k-NN: k-Nearest Neighbors algorithm; SVM: Support Vector Machine; ResNet: Residual Neural Network.

**Table 2:** Summary of AI-enabled DTs in smart manufacturing: **machinery and equipment level** (Section 2.3).

Subfield	AI-Category	Key Methods	Application-Case	Ref.
Condition Monitoring (Section 2.3.2.1)	Supervised Learning	ANN	Prediction of process forces	[133]
		CNN	Prediction of process forces	[134]
		CNN, SVDD	Defect recognition of steel surfaces	[135]
		CNN-DLSTM based transfer learning	Fault detection of rolling bearings	[136]
	Unsupervised Learning	GAN	Prediction of machining vibration signals	[137]
		Dictionary learning, transfer learning	Wave field prediction for damage detection with ultrasonic guided wave	[138]
	Computational Intelligence	Fuzzy inference	Brake CM of an overhead crane	[139]
Predictive Maintenance (Section 2.3.2.2)	Supervised Learning	PGM, MCMC	Prediction of stress-intensity factors and RUL	[140]
		RCM	Prediction of RUL of a drilling machine	[141]
		RF, particle filter	Prediction of tool wear	[142]
		Deep Stacked GRU	Prediction of tool wear	[143]
		LSTM	Equipment utilization prediction	[144]
		LSTM	Tool condition prognostic model	[145]
		LSTM	Estimation of RUL of the machine components	[146]
	Unsupervised Learning	GMM	Tool failure prediction	[147]
		SSAE-PHMM	Prediction of tool wear	[148]
		SSAE, deep transfer learning	Fault prognosis in a car body-side production line	[149]
		GAN, VAE	Generation of a health indicator for PHM of rotating systems	[150]
		CAE	Construction of a health indicator for bearings	[151]
		Distributed k-means	Assessing MAS for collaborative PdM	[152]
	Computational Intelligence	Bayesian network	Mission planning under uncertainty with respect to fatigue cracking	[153]
Dynamics & Control (Section 2.3.2.3)	Supervised Learning	RNN	Prediction of dynamic states in metal cutting	[154]
		ANN	Prediction of resonances frequencies of a thin bulk acoustic wave resonator	[155]
	Computational Intelligence	Gaussian process	Estimation of single-degree-of-freedom dynamic systems	[156]
		Gaussian process	Prediction of the dynamic response	[157]
		GWO	Optimization of motion control system in machine tools	[158]

SVDD: Support Vector Data Description; RCM: Random Coefficient Model; RF: Random Forest; MCMC: Markov Chain Monte Carlo; GRU: Gated Recurrent Units; (D)LSTM: (Deep) Long Short Term Memory; GMM: Gaussian Mixture Model; GAN: Generative Adversarial Network; SSAE-PHMM: Stack Sparse AutoEncoder Parallel Hidden Markov Model; VAE: Variational AutoEncoder; CAE: Convolutional AutoEncoder; MAS: Multi-Agent System; RNN: Recurrent Neural Network; GWO: Grey Wolf Optimization.

**Table 3:** Summary of AI-enabled DTs in smart manufacturing: **process and material level** (Section 2.4).

Subfield	AI-Category	Key Methods	Application-Case	Ref.
Metal Cutting (Section 2.4.2.1)	Supervised Learning	PIO, SVM	Prediction of surface roughness	[195]
		Ensemble methods, ANN	Modeling of the rheological behavior of drilling fluids	[196]
		ANN	Prediction of stress and fatigue damage (FE surrogate) of flexible risers	[197]
		DNA-based computing, Markov chain	Prediction of surface roughness	[198]
	Reinforcement Learning	DDPG	Optimization of decision-making based on performance and machinability of parts	[199]
	Computational Intelligence	PSO	Inverse determination of material model parameters from cutting simulation	[200]
Metal AM and Laser Material Processing (Section 2.4.2.2)	Supervised Learning	SVM	Prediction of the occurrence of defects in metal AM (LPBF, LMD)	[201]
		CNN, LSTM, RNN	Quality assurance in metal AM (LPBF)	[202]
		CART	Prediction of additive manufacturability	[203]
		HMM	Model adaptivity and quality assessment of laser material removal processes	[204]
	Unsupervised Learning	k-means	Anomaly detection and process optimization of 3D laser cutting processes	[205]
Composite Material Processing (Section 2.4.2.3)	Supervised Learning	CNN, transfer learning	Detection of dry points in the production of carbon fiber reinforced plastics	[206]
		AdaBoost, XGBoost, RF	Prediction of temperature distribution of thermoplastic composites	[207]
		DNN	FE surrogate for a composite textile draping process	[208]
		PML	Prediction of material properties of a composite material system	[209]
	Computational Intelligence	ISRES	Identification of material parameters of a prepreg sheet	[210]
Joining	Supervised Learning	DNN, GA	Prediction of distortion in welding	[211]
Forming	Supervised Learning	ANN	Prediction of the ingate velocity during sand mold filling	[212]

PIO: Pigeon-Inspired optimization; DDPG: Deep Deterministic Policy Gradient; PSO: Particle Swarm Optimization; HMM: Hidden Markov Model; PML: Probabilistic Machine Learning; ISRES: Improved Stochastic Ranking Evolution Strategy.

**Table 4:** Summary of AI-enabled DTs in advanced robotics.

Subfield	AI Category	Key Methods	Application-Case	Ref.
Control (Section 3.2)	Supervised and Unsupervised Learning	SVM, PCA	Object recognition of a smart gripper	[260]
	Reinforcement learning	Trial-and-error search	Weightlifting robot control	[261]
	Supervised Learning	GD	Understanding the added value of integrated models for through-life engineering services	[262]
	Computational Intelligence	Vision-based Markovian chain	Automate fan-blade reconditioning for aerospace maintenance, repair and overhaul	[263]
		QP	Supporting rescuers on disaster-response missions	[264]
Planning (Section 3.3)	Reinforcement Learning	Proximal policy optimization	Pick-and-place tasks for an industrial robotic arm	[265]
		DDPG	Control and trajectory planning of a planar 3-DOF manipulator and 3D arms of a humanoid robot	[266]
		DQN	Automate smart manufacturing systems	[267]
		Proposed LSTM-MACG	Collision avoidance for a number of UAVs in a confined airspace	[268]
	Computational Intelligence	Ant colony optimization	Path planning of industrial robots	[269]
HRI/HRC (Section 3.4)	Supervised Learning	CNN	Standing-posture recognition in HRC	[270]
		DL	Mechatronics system	[271]
		ANN	Enabling industrial robots to bypass obstacles	[272]
		LSTM	Visual question answering for HMC system	[273]
	Supervised and Unsupervised Learning	FFT-PCA-SVM	HRI welding and welder behavior analysis (identifying the professional level)	[274]
	Reinforcement Learning	DDPG	COVID-19, improve efficiency in assembling medical equipment	[275]
Predictive Maintenance	Supervised Learning	DNN	System health monitoring	[276]
			Maximizing the overall plant availability of modern manufacturing systems	[277]
Workspace Modeling	Supervised Learning	Monte Carlo method	Simulating the workspace of the mechanisms	[278]
Others	Supervised Learning	RF	Estimation of lawn grass lengths for robotic lawn mower	[279]

PCA: Principal Component Analysis; GD: Gradient Descent; QP: Quadratic Programming; MACG: MultiAgent Computational Guidance; FFT: Fast Fourier Transform; UAV: Unmanned Aerial Vehicle.

**Table 5:** **Summary of AI-enabled DTs in sustainable development and FESG factors**.

F-Factor	E-Factor	SG-Factor
Productivity (Section 2.2)	Production Planning (Section 2.2.2.1)	Intelligent Sensing of Novel Indicators (Section 2.2.2, Section 2.3.2, and Section 2.4.2)
	Production Control (Section 2.2.2.2)	
	Quality Control (Section 2.2.2.3)	
Availability (Section 2.3)	Condition Monitoring (Section 2.3.2.1)	Innovative Robot Planning and Control (Section 3.2 and Section 3.3)
	Predictive Maintenance (Section 2.3.2.2)	
	Dynamics and Control (Section 2.3.2.3)	HRI/HRC (Section 3.4)
	Robot Maintenance (Section 3.5)	
Quality (Section 2.4)	Metal Cutting (Section 2.4.2.1)	DfX (Section 2.4.2.1 and Section 2.4.2.2)
	Metal Additive Manufacturing (Section 2.4.2.2)	XaaS and Business Model (Section 2.2.3)
	Composite Material Processing (Section 2.4.2.3)	

## Data Availability

Not applicable.

## Abbreviations

The following abbreviations are used in this manuscript:

AdaBoost	Adaptive Boosting
AI	Artificial Intelligence
AM	Additive Manufacturing
ANN	Artificial Neural Network
AR	Augmented Reality
CAD	Computer-Aided Design
CAE	Convolutional AutoEncoder
CAM	Computer-Aided Manufacturing
CAQ	Computer-Aided Quality Assurance
CART	Classification and Regression Tree
CM	Condition Monitoring
CNC	Computer Numeric Control
CNN	Convolutional Neural Network
CRM	Customer Relationship Management
DBN	Dynamic Bayesian Network
DDPG	Deep Deterministic Policy Gradient
DES	Discrete Event Simulation
DfX	Design for X
DL	Deep Learning
DNN	Deep Neural Network
DOF	Degrees of Freedom
DQN	Deep Q-Network
DT	Digital Twin
ERP	Enterprise Resource Planning
FE	Finite Element
FFT	Fast Fourier Transform
GA	Genetic Algorithm
GAN	Generative Adversarial Network
GCN	Graph Convolution Network
GD	Gradient Descent
GMM	Gaussian Mixture Model
GP	Gaussian Process
GPT	General-Purpose Technology
GPU	Graphical Processing Unit
GRU	Gated Recurrent Units
GWO	Grey Wolf Optimization
HMC	Human-Machine Collaboration
HMM	Hidden Markov Model
HPC	High-Performance Computing
HRC	Human-Robot Collaboration
HRI	Human-Robot Interaction
IoT	Internet of Things
ISRES	Improved Stochastic Ranking Evolution Strategy
k-NN	k-Nearest Neighbors algorithm
KPI	Key Performance Indicators
LMD	Laser Melting Deposition
LPBF	Laser Powder Bed Fusion
LSTM	Long Short-Term Memory
MAS	Multi-Agent System
MBSE	Model-Based System Engineering
MCMC	Markov Chain Monte Carlo
MDP	Markov Decision Process
MES	Manufacturing Execution System
ML	Machine Learning
NASA	National Aeronautics and Space Administration
NIST	National Institute of Standards and Technology
NN	Neural Network
PCA	Principal Component Analysis
PDA	Production Data Acquisition
PdM	Predictive Maintenance
PGM	Probabilistic Graphical Model
PHM	Prognostics and systems Health Management
PIO	Pigeon-Inspired Optimization
PLM	Product Lifecycle Management
PML	Probabilistic Machine Learning
PPC	Production Planning and Control
PSO	Particle Swarm Optimization
QP	Quadratic Programming
RCM	Random Coefficient Model
ResNet	Residual Neural Network
RF	Random Forest
RL	Reinforcement Learning
RNN	Recurrent Neural Network
RTM	Resin Transfer Molding
RUL	Remaining Useful Life
SCM	Supply Chain Management
SME	Small and Medium-sized Enterprise
SSAE-PHMM	Stack Sparse AutoEncoder Parallel Hidden Markov Model
SVDD	Supported Vector Data Descriptor
SVM	Support Vector Machine
TRPO	Trust Region Policy Optimization
UAV	Unmanned Aerial Vehicle
VR	Virtual Reality
VUCA	Volatile, Uncertain, Complex, Ambiguous
WGP	German Academic Society for Production Engineering
XaaS	X-as-a-Service
